# Transparent and conducting boron doped ZnO thin films grown by aerosol assisted chemical vapor deposition†

**DOI:** 10.1039/d2ra05895b

**Published:** 2022-11-17

**Authors:** Donglei Zhao, Sanjayan Sathasivam, Mingyue Wang, Claire J. Carmalt

**Affiliations:** Materials Chemistry Centre, Department of Chemistry, University College London 20 Gordon Street London WC1H 0AJ UK; School of Engineering, London South Bank University London, SE1 0AA UK c.j.carmalt@ucl.ac.uk +44 20-7679-7463

## Abstract

ZnO based transparent conducting oxides are important as they provide an alternative to the more expensive Sn : In_2_O_3_ that currently dominates the industry. Here, we investigate B-doped ZnO thin films grown *via* aerosol assisted chemical vapour deposition. B : ZnO films were produced from zinc acetate and triethylborane using either tetrahydrofuran or methanol (MeOH) as the solvent. The lowest resistivity of 5.1 × 10^−3^ Ω cm along with a visible light transmittance of ∼75–80% was achieved when using MeOH as the solvent. XRD analysis only detected the wurtzite phase of ZnO suggesting successful solid solution formation with B^3+^ substituting Zn^2+^ sites in the lattice. Refinement of the XRD patterns showed minimal distortion to the ZnO unit cell upon doping when MeOH was the solvent due to the immiscibility of the [BEt_3_] solution (1.0 M solution in hexane) in methanol that limited the amount of B going into the films, thus preventing excessive doping.

## Abstract 2

Boron doped zinc oxide thin films *via* aerosol assisted chemical vapor deposition with resisitivities as low as 5.1 × 10^^−3^^ Ω cm.

## Graphical abstract

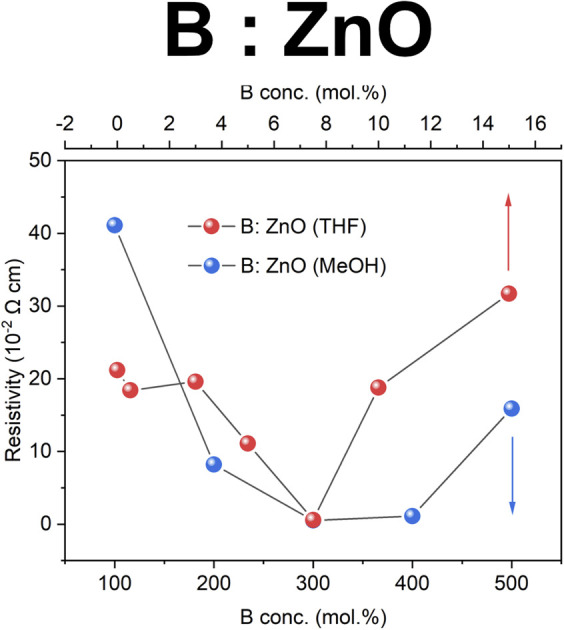

## Introduction

Transparent conducting oxides (TCOs) are vital semiconductor materials widely used in many areas, such as screen displays, touchscreens, solar cells, LCD panels and OLEDs.^1–4^ They have a wide band gap that allows for visible light transparency and relatively high carrier concentration (∼≥×10^20^ cm^−3^) that enables low electrical resistivity.^1,3,5^ The high carrier concentration arises due to a combination of intrinsic impurities and extrinsic dopants.^6–8^

Currently, tin doped indium oxide (ITO) and fluorine doped tin oxides (FTO) are the most widely used TCO materials due to their high performance *i.e.*, resistivities ≤5 × 10^−4^ Ω cm and transparencies >80%.^2,3,8–11^ However, ITO is fast becoming financially unviable due to the increasing cost of In whilst FTO suffers from intrinsic limitations that has meant further enhancement in optoelectronic performance is unattainable.^12^ Research into new TCOs is needed to find potential replacements for ITO and FTO.^5,13–17^

TCOs based on ZnO have the potential to become leading players due to the high abundance of Zn and low cost.^18,19^ The band gap of ZnO is ∼3.37 eV leading to high transmittance.^20,21^ Traditionally, ZnO is doped with Group 13 ions such as Al^3+^ or Ga^3+^ as their higher valence and acceptable ionic radii allow for an increase in carrier concentration without massive distortion of the ZnO lattice.^7,13,14^ Typically resistivities as low as 5.6 × 10^−4^ Ω cm^22^ and 5.0 × 10^−3^ Ω cm^7^ have been achieved *via* both physical^22,23^ and chemical vapor deposition^7^ routes.

ZnO can also be doped substitutionally on Zn^2+^ sites with boron in the +3 oxidation state to enhance carrier concentration and increase conductivity. B^3+^ has a smaller ionic radius compared to Zn^2+^ and is highly soluble in ZnO. Furthermore, the enthalpy of formation of B_2_O_3_ (−13.18 eV) is higher than that of Al_2_O_3_ (−17.37 eV) therefore suggesting unwanted secondary oxide phases that can negatively impact the conductivity are less likely to form when B is used as a dopant compared to Al.^24,25^ For example, Lu *et al.* found for sputtered Al : ZnO films at higher concentrations of dopant, possible cluster and precipitate formation within and on the boundaries of grains caused detrimental film properties including a reduction in the conductivity, carrier concentration and mobility.^26^ This may possibly be due to the formation of the thermodynamically favorable amorphous Al_2_O_3_ phase.

B : ZnO thin films have been grown *via* magnetron sputtering,^27^ metal organic chemical vapor deposition (MOCVD),^28^ spray deposition^29^ and sol–gel methods^30^ yielding resistivities as low as 7.5 × 10^−3^ Ω cm,^27^ 10 Ω/⧠,^28^ 4.5 × 10^−3^ Ω cm,^29^ and 2.2 × 10^2^ Ω cm.^30^ B : ZnO has been shown to be particularly good for increasing the efficiency of Cu(In, Ga)Se_2_ (CIGS) photovoltaics.^31^

In this paper, we use a specialized form of CVD called aerosol assisted (AA) CVD that allows the growth of transparent and conducting B : ZnO films using non-volatile, commercially available and inexpensive precursors, namely zinc acetate hydrate and triethylborane. AACVD is unique in that the CVD precursors are dissolved in an appropriate solvent to form a solution that is transferred to the vapor phase in the form of aerosol droplets using a pizoelectronic device. The aerosol mist is then moved into the CVD growth chamber using a carrier gas. The AACVD method is advantageous as it enables device quality films under scalable ambient pressure conditions.^6,8,11,32–35^ AACVD has been used to prepare many thin films widely used in different areas such as for photovoltaics, sensors and photocatalysis.^36–38^ Here, a series of B : ZnO thin films from two different solvents (THF and methanol) have been prepared on glass substrates *via* AACVD and their material and optoelectronic characteristics tested. It was found that resistivities as low as 5.8 × 10^−3^ Ω cm for THF as the solvent and 5.1 × 10^−3^ Ω cm for MeOH as the solvent were obtainable with visible light transparency of ∼75–90% for all the thin films.

## Experimental

### Film synthesis

Depositions were carried out under N_2_ (BOC Ltd., 99.99% purity) flow. Zinc acetate dihydrate (Zn(OAc)_2_·2H_2_O), triethylborane (BEt_3_) solution (1.0 M in hexanes), acetone (99%), tetrahydrofuran (THF, 99%) and methanol (MeOH, 99%) were purchased from Sigma. Glass substrates were cleaned using detergent, water and isopropanol then dried in a 70 °C oven.

For the B : ZnO thin films from THF solvent, Zn(OAc)_2_·2H_2_O (0.40 g, 1.82 mmol) in THF (20 mL) was placed in a glass bubbler. [BEt_3_] at 0, 0.5, 3, 7.5, 10 and 15 mol% of Zn(OAc)_2_·2H_2_O was added to the same bubbler.

For the B : ZnO thin films from MeOH solvent, Zn(OAc)_2_·2H_2_O (0.40 g, 1.82 mmol) in MeOH (20 mL) was placed in a glass bubbler with [BEt_3_] at 0, 100, 200, 300, 400 and 500 mol% of Zn(OAc)_2_·2H_2_O also added. Several drops of acetone were also added to the bubbler to aid dissolution of the precursors.

All solutions were atomised using a piezoelectric device (Johnson Matthey liquifog^®^). The aerosol mist was delivered to the AACVD reaction chamber and passed over the heated substrate (float glass with a SiO_2_ barrier layer) using N_2_ carrier gas at 1.0 L min^−1^.^39^ Depositions were carried out at 475 °C and lasted until the precursor solution was fully used. After the depositions the substrates were cooled under a flow of N_2_. The glass substrates would not be removed unless that with the graphite block was cooled to below 50 °C. The films on the substrates were handled and stored in air.

### Film characterisation

The X-ray diffraction (XRD) analysis scanning from 10 to 65° (2*θ*) used a modified Bruker-Axs D8 diffractometer with parallel beam optics and a PSD LynxEye silicon strip detector. The scans used a monochromated Cu Kα source operated at 40 kV and its emission current was 30 mA with 0.5° as incident beam angle and 0.05° at 1 s per step as step frequency. The JEOL JSM-6301F Field Emission Scanning Electron Microscopy (SEM) with 5 keV as accelerating voltage was used to investigate the surface morphologies of the thin films. To avoid charging, all the samples were coated with gold before the analysis. The X-ray photoelectron spectroscopy (XPS) analysis was used to determine the surface elemental surroundings by a Thermo Scientific K-alpha photoelectron spectrometer using monochromatic Alk_α_ radiation. Higher resolution scans were recorded for the principal peaks of zinc (Zn 2p), boron (B 2s), oxygen (O 1s) and carbon (C 1s) at a pass energy of 50 eV, and then the CasaXPS software was used to deal with the data from the XPS analysis. The binding energy of adventitious carbon was adjusted at 284.5 eV as calibration. The Filmetrics F20 thin-film analyzer was used to measure the thickness of thin films optically using reflectance spectroscopy. The optical properties were determined through a PerkinElmer Fourier transform Lambda 950 spectrometer scanning between 2500 nm and 300 nm. Hall effect measurements were used to determine the of the films resistivity (*ρ*) *via* the van der Pauw method with a permanent magnet (0.58 T) and one constant current (1 mA, 1 μA).

## Results and discussion

B-doped ZnO thin films were prepared from zinc acetate dihydrate (Zn(OAc)_2_·2H_2_O) as Zn precursor and when necessary, triethylborane solution ([BEt_3_], 1.0 M in hexanes) as the B dopant source *via* AACVD. Two solvents, tetrahydrofuran (THF) and methanol (MeOH) were studied due to the differing solubility/immiscibility of the Zn and B precursors in each solvent. Zn(OAc)_2_·2H_2_O is highly soluble in MeOH but less soluble in THF whereas the [BEt_3_] solution in hexane is miscible in THF but immiscible in MeOH. Therefore, larger mol% of the [BEt_3_] solution in hexane were added when MeOH was the solvent. All depositions were carried out at a substrate temperature of 475 °C and N_2_ flow rate of 1.0 L min^−1^ to allow for optimal substrate coverage, ZnO crystallinity and film thickness. All the B : ZnO thin films were well adhered to the substrate and passed the Scotch tape test.^8^

### X-ray diffraction

**Fig. 1:**
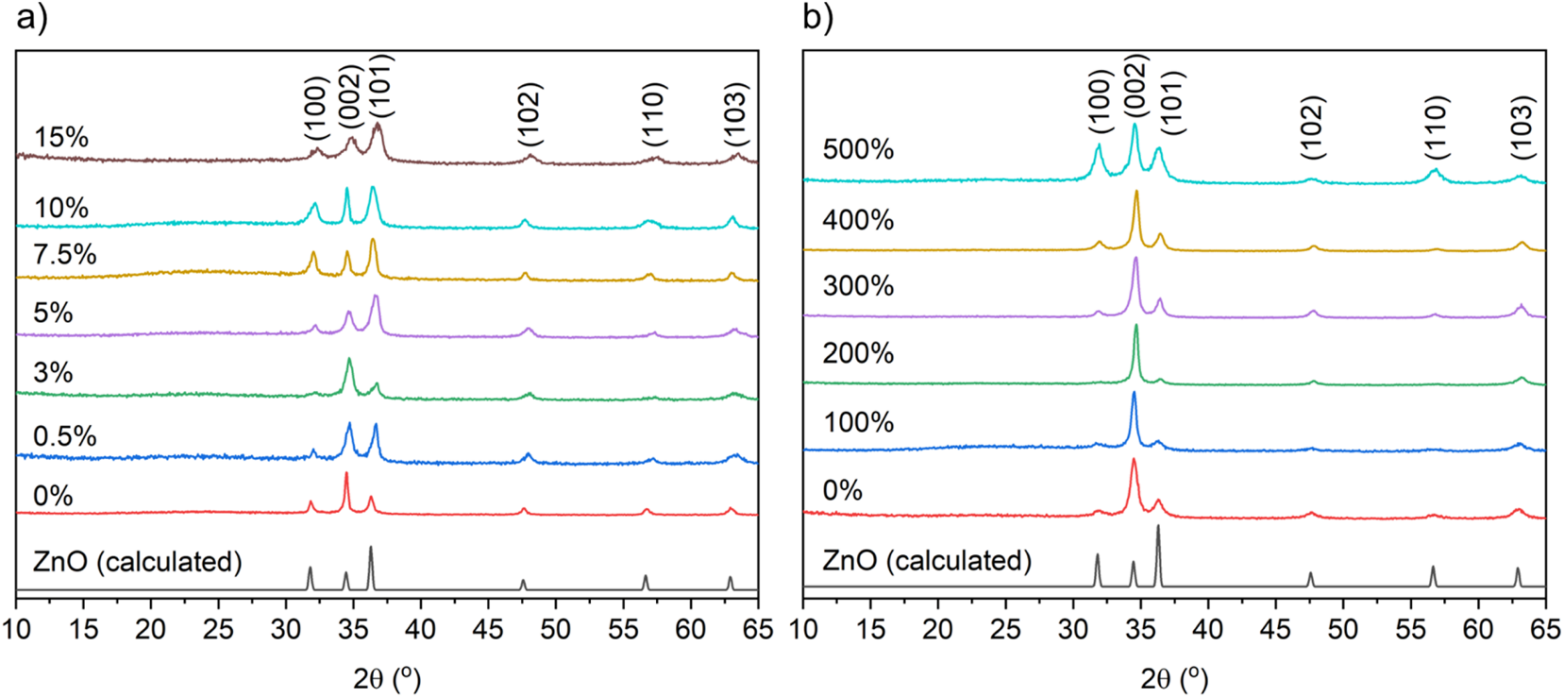
XRD patterns showing the undoped and B-doped ZnO films using (a) THF and (b) MeOH as solvents prepared at 475 °C to be in the wurtzite phase of ZnO.

The X-ray diffraction patterns of the undoped and B-doped ZnO films from THF and MeOH are illustrated in Fig. 1. The detected peaks at 31.8, 34.4, 36.3, 47.5, 56.6 and 63.0° correspond to (100), (002), (101), (102), (110) and (103) planes of the expected wurtzite phase of ZnO. No peaks for any secondary phases were visible suggesting successful solid solutions had been formed. Texture coefficients were calculated for the ZnO films to determine the extent of preferential orientation of the crystallographic planes (ESI†).^40^ For the nominally undoped and B : ZnO thin films from THF and MeOH (Fig. 1) preferred orientation was observed in the (002) plane, which is expected as this is the lowest surface energy plane (see ESI Fig. S1 and 2†) and therefore most likely to dominate.^41^

Tables 1 and 2 show the unit cell parameters for the undoped and B-doped ZnO films from THF and MeOH solutions as determined from Le Bail refinement of the powder XRD data. Interestingly, a general decrease in the ZnO unit cell volume from 47.50(2) Å^3^ for the 0 mol% to 45.86(22) Å^3^ at 15 mol% for the films gown using THF was observed. This is due to the smaller B^3+^ (0.23 Å) ions substituting for the larger Zn^2+^ (0.60 Å) ions resulting in a unit cell contraction.^13,42^ With MeOH as the solvent, where solubility of Zn(OAc)_2_·H_2_O is high and the [BEt_3_] solution is immiscible, no change in the ZnO unit cell volume was observed upon doping. This is likely due to the very low concentrations of B actually doping into the film therefore minimizing the distortion caused to the ZnO lattice.

**Table 1:** The unit cell parameters for the pure ZnO and B doped ZnO thin films from THF solvent grown *via* AACVD

*B*/mol%	Unit cell parameters		
	*a*/Å	*c*/Å	Volume/Å^3^
0	3.248(1)	5.198(1)	47.50(2)
0.5	3.215(5)	5.174(5)	46.30(11)
3	3.216(15)	5.166(6)	46.28(31)
5	3.210(3)	5.176(4)	46.19(7)
7.5	3.228(5)	5.203(7)	46.94(12)
10	3.226(6)	5.197(5)	46.84(13)
15	3.201(10)	5.170(10)	45.86(22)

### X-ray photoelectron spectroscopy

**Fig. 2:**
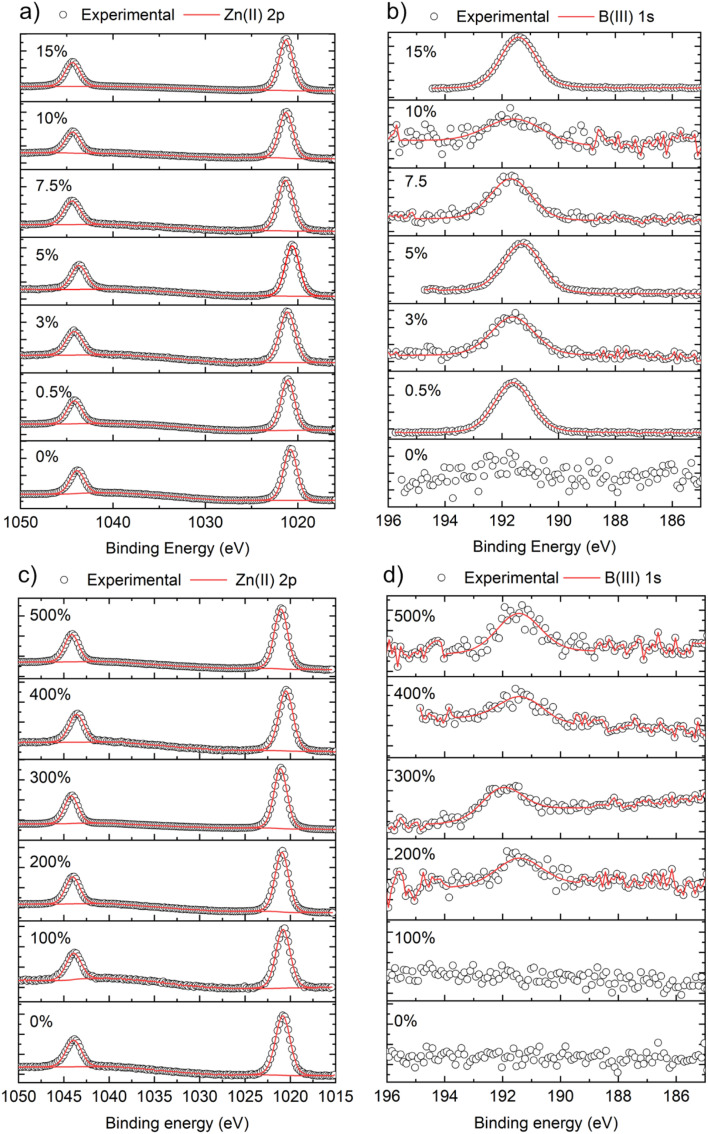
Core level XPS analysis showing the surface compositions and oxidation of the undoped and B-doped ZnO thin films using (a and b) THF and (c and d) MeOH as solvents and grown *via* AACVD.

X-ray photoemission spectroscopy (XPS) was carried out to determine the surface composition and oxidation state of the B : ZnO films, as shown in Fig. 2. For all films, the Zn 2p_3/2_ peaks were centered at ∼1020.6 eV which correspond to Zn^2+^ (Fig. 2a and c).^43^ For films grown using THF, the B 1s peaks (when detected) were centered at ∼191.6 eV, which corresponds to B in the expected 3+ oxidation state.^18^ For the films grown using MeOH as the solvent, the signal to noise ratio for the B 1s was low compared to the THF samples, again providing more evidence for the low concentration of B in these ZnO films, as also suggested by the XRD data (Table 2). In fact, no B was detected even when 100 mol% of [BEt_3_] was used in the MeOH solution.

**Table 2:** The unit cell parameters for the pure ZnO and B doped ZnO thin films from MeOH solvent grown *via* AACVD

*B*/mol%	Unit cell parameters		
	*a*/Å	*c*/Å	Volume/Å^3^
0	3.248(4)	5.201(1)	47.51(8)
100	3.249(6)	5.197(0.9)	47.51(12)
200	3.240(10)	5.177(4)	47.07(21)
300	3.241(4)	5.179(2)	47.12(8)
400	3.239(6)	5.171(2)	46.99(12)
500	3.243(2)	5.187(2)	47.26(5)

### Scanning electron microscopy

**Fig. 3:**
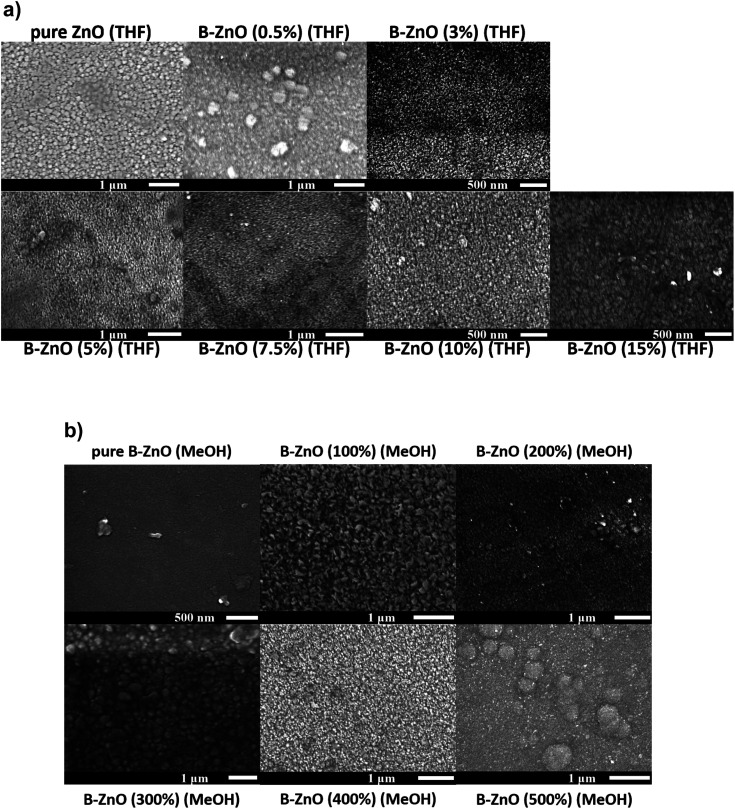
SEM images for the morphology of the undoped and B doped ZnO thin films using THF (a) and MeOH (b) as solvents prepared through AACVD.

Fig. 3 shows the surface morphologies of the ZnO films with a series of B concentrations grown using THF and MeOH as solvents. The morphology of the ZnO based thin films may be influenced by several factors including the solvent, precursor, oxidant source, substrate, carrier gas and deposition temperature.

For both the ZnO based films deposited using THF and MeOH as solvents, the nominally undoped ZnO film consisted of randomly oriented grains in varying sizes, similar to what has previously been seen for CVD grown ZnO.^13^ As B was introduced into the films, minimal impact on the morphology was observed for the films grown from THF solutions, however when MeOH was used as the solvent the presence of the B dopant caused a more noticeable change in the surface morphology. Previous reports have described the influence that MeOH can have on the microstructure of thin films deposited *via* AACVD, and in general aerosols from different solvents can influence the microscopic surface morphology besides their normal transportation effect.^44,45^ Therefore, the variations observed in the morphology of the films in this study is consistent with literature.

### UV-visible-near infrared spectroscopy

**Fig. 4:**
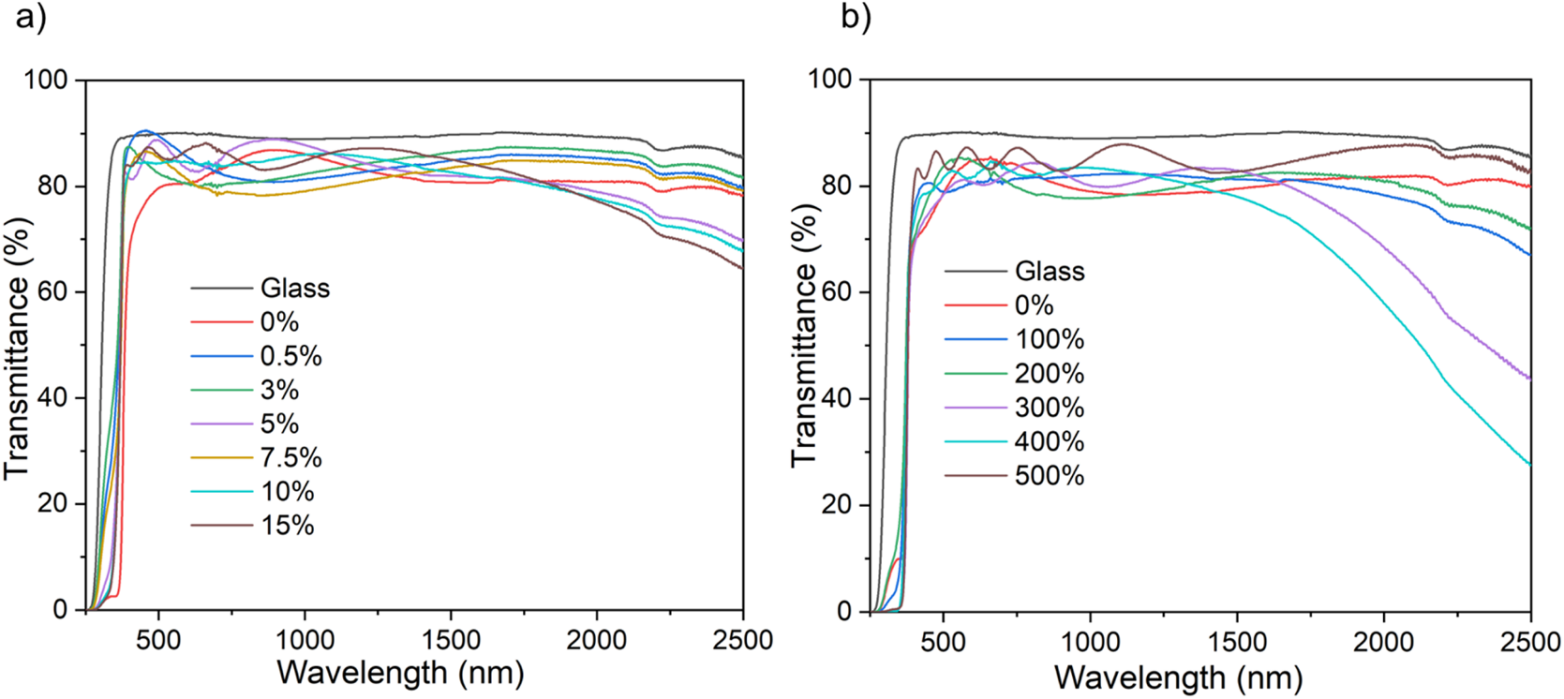
The optical data for the undoped and B doped ZnO thin films on glass substrates using THF (a) and MeOH (b) as solvents prepared *via* AACVD showing the UV/vis spectra.

The optical property of the films, namely transmittance, has been determined using ultraviolet-visible-near infrared spectroscopy (UV-vis-NIR) (Fig. 4). All the B : ZnO thin films regardless of solvent used for the AACVD, showed transmittance between ∼75–90% in the visible range – making them suitable for TCO application. The film thickness, as determined *via* reflectance UV-vis spectroscopy using a Filmetrics instrument, increased with increasing amount of [BEt_3_] used in the precursor solution (See ESI Tables S1 and S2†). This is attributed to interactions between the zinc acetate and the [BEt_3_], either in solution in the bubbler or in the gas phase in the CVD chamber and the formation of intermediate products that decomposed more efficiently to give B : ZnO films.

In the near infrared area, a decrease in transmittance was observed with increasing B concentration for both solvent systems though this was more pronounced for the MeOH samples. This is associated with the increase in free carrier concentration caused by B^3+^ substitution that leads to an increase in the plasmon resonance frequency from the NIR towards the visible.^6,13,46^

### Hall effect measurements

The resistivities of the B : ZnO films were measured *via* the Hall effect measurement while the parameter film thickness was calculated through the reflectance spectroscopy, as given in Tables S1 and S2 in ESI.† The nominally undoped film deposited using MeOH was too resistive to obtain any values but crude measurements *via* a two-point probe showed the resistance to be in the MΩ order. For the THF solvent system, the nominally undoped film was measurable but still high at 2.12 × 10^−1^ Ω cm. The differences observed may be due to intrinsic vacancies/dopants such as oxygen vacancies, zinc interstitials or even adventitious hydrogen.^47^

**Fig. 5:**
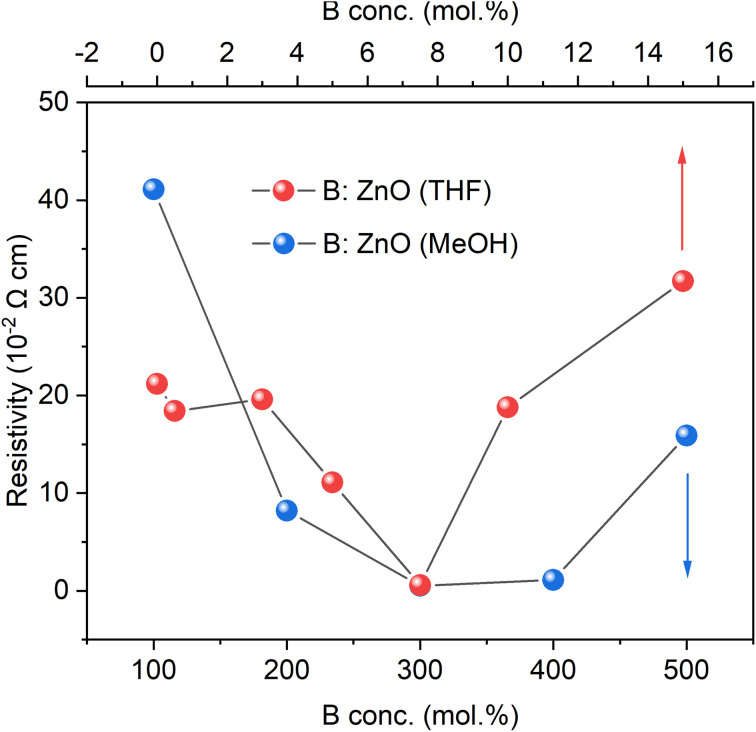
The resistivities of the undoped and B doped ZnO films using THF and MeOH as solvents grown through AACVD derived from Hall measurements.

For both systems, an increase in the B concentration caused a decrease in resistivity is likely due to an increase in the carrier concentration (as also observed in the UV-vis-NIR spectra as a decrease in the NIR transmittance). The lowest resistivity of 5.8 × 10^−3^ Ω cm for THF as the solvent and 5.1 × 10^−3^ Ω cm for the MeOH based films were achieved using 7.5 and 300 mol% of [BEt_3_] in the AACVD solution respectively. According to the significant difference in miscibility for the B source ([BEt_3_] solution in hexane) in THF (high) and MeOH (low), the initial B concentrations of 7.5 mol% with THF as solvent and 300 mol% with MeOH as solvent were adopted in order to achieve similar bulk B concentrations (at%) after depositions, close to the B solubility limit in ZnO material leading to lowest resistivities. The resulting low resistivities data for these films was achieved using the abundant B dopants and are comparable to typical TCOs material, Al : ZnO^48^ thin films grown from the same synthesis technology (AACVD) and the same Zn source (Zn(OAc)_2_·2H_2_O) with resistivities of 3.54 × 10^−3^ Ω cm.^48^ B : ZnO thin films also have been investigated as TCOs materials from some other synthetic routes, such as radio frequency magnetron sputtering^18^ and chemical spray pyrolysis^29^ and their lowest resistivities were 5.65 × 10^−3^ Ω cm and 4.5 × 10^−3^ Ω cm, respectively, which are similar to the lowest resistivities in this study although here the scalable and inexpensive synthesis method of AACVD was used (Fig. 5).

## Conclusion

B-doped ZnO films were grown using Zn(OAc)_2_·2H_2_O and dopant quantities of [BEt_3_]. Two solvents were studied, THF and MeOH, due to the differing solubilities/miscibility of the precursors and to investigate the effect of the change in solvent system on the film properties. XRD alluded to the successful solid solution formation involving substitution of B^3+^ on Zn^2+^ sites in the ZnO lattice. Furthermore, XPS studies showed that B was indeed in the 3+ oxidation state thus donating one electron for conduction for every Zn^2+^ substituted. An increase in carrier concentration resulted in reduced transmittance in the NIR region of the UV-vis-NIR spectra for the doped samples. This was more pronounced in the MeOH samples compared to THF therefore suggesting that the formers carrier concentration was higher. The lowest resistance of 5.1 × 10^−3^ Ω cm was achieved for the 300 mol% [BEt_3_] using MeOH as the solvent.

## Conflicts of interest

The authors have no conflicts of interest to declare.

## Supplementary Material

RA-012-D2RA05895B-s001
