# Supplementary material for: Transparent and conducting boron doped ZnO thin films grown by aerosol assisted chemical vapor deposition
Source: RSC Adv. 2022 Nov 17;12(51):33049–55. doi: 10.1039/d2ra05895b (PMC9671099; doi:10.1039/d2ra05895b)
Supplement: RA-012-D2RA05895B-s001 [file RA-012-D2RA05895B-s001.pdf]

Electronic Supplementary Material (ESI) for RSC Advances.
This journal is © The Royal Society of Chemistry 2022

Supporting Information

Transparent and Conducting Boron Doped ZnO Thin Films Grown By Aerosol Assisted Chemical Vapor Deposition

Donglei Zhao[a], Sanjayan Sathasivam[a,b], Mingyue Wang[a] and Claire J. Carmalt[a]*

*Corresponding author

(a) Materials Chemistry Centre, Department of Chemistry, University College London, 20 Gordon Street, London WC1H 0AJ, UK

(b) School of Engineering, London South Bank University, London, SE1 0AA

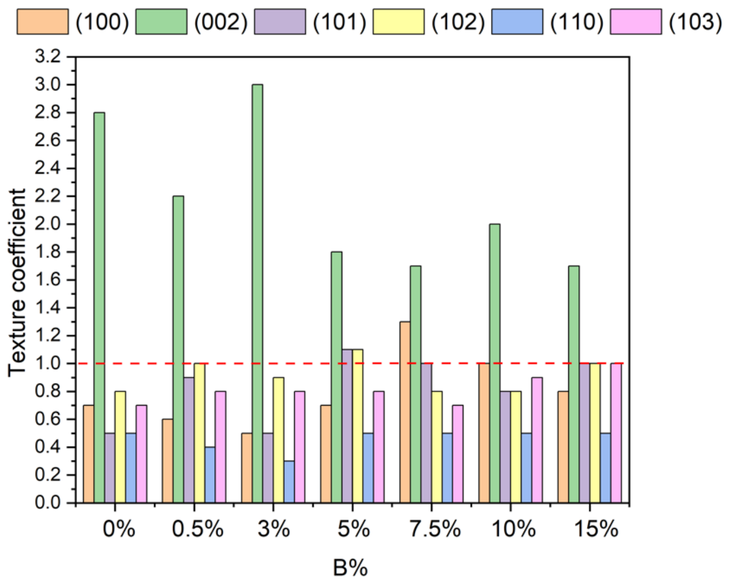

Figure S1: Texture coefficient determined from the XRD data for pure and B:ZnO films grown using THF as the solvent via an AACVD process.

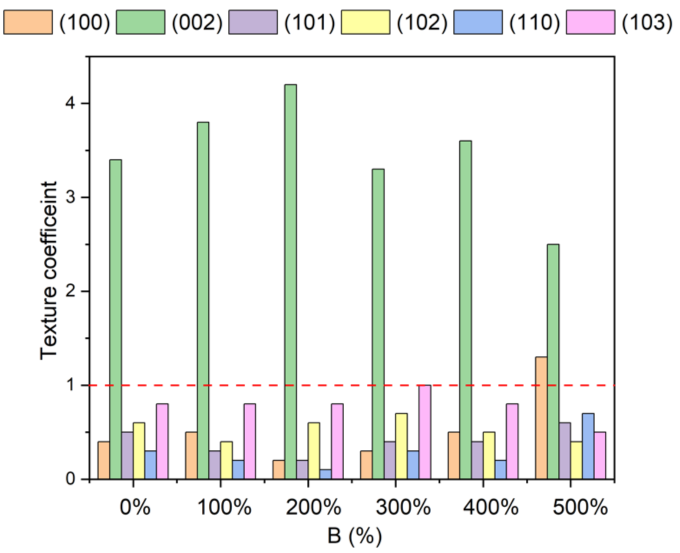

Figure S2: Texture coefficient determined from the XRD data for pure and B:ZnO films grown using methanol as the solvent via an AACVD process.

Table S1: The film thickness from Filmetrics measurement for the ZnO:B thin films from THF solvent grown at 475 ^{o}C through AACVD

Initial B concentrations (mol.%)	Film thickness (nm)
0	180
0.5	150
3	250
5	310
7.5	200
10	480
15	450

Table S2: The film thickness from Filmetrics measurement for the ZnO:B thin films from MeOH solvent grown at 475 ^{o}C through AACVD

Initial B concentrations (mol.%)	Film thickness (nm)
0	250
100	210
200	190
300	230
400	840
500	530
